# Cortical and behavioral tracking of rhythm in music: Effects of pitch predictability, enjoyment, and expertise

**DOI:** 10.1111/nyas.15315

**Published:** 2025-03-18

**Authors:** Anne Keitel, Claire Pelofi, Xinyi Guan, Emily Watson, Lucy Wight, Sarah Allen, Iris Mencke, Christian Keitel, Johanna Rimmele

**Affiliations:** ^1^ Department of Psychology University of Dundee Dundee UK; ^2^ Department of Psychology New York University New York New York USA; ^3^ Max Planck NYU Center for Language, Music, and Emotion New York New York USA; ^4^ Digital and Cognitive Musicology Lab École Polytechnique Fédérale de Lausanne Lausanne Switzerland; ^5^ School of Psychology Aston University Birmingham UK; ^6^ Department of Medical Physics and Acoustics University of Oldenburg Oldenburg Germany; ^7^ Department of Music Max‐Planck‐Institute for Empirical Aesthetics Frankfurt Germany; ^8^ Department of Cognitive Neuropsychology Max‐Planck‐Institute for Empirical Aesthetics Frankfurt Germany

**Keywords:** EEG, music perception, musical expertise, naturalistic music, pitch surprisal, top‐down influences

## Abstract

The cortical tracking of stimulus features is a crucial neural requisite of how we process continuous music. We here tested whether cortical tracking of the beat, typically related to rhythm processing, is modulated by pitch predictability and other top‐down factors. Participants listened to tonal (high pitch predictability) and atonal (low pitch predictability) music while undergoing electroencephalography. We analyzed their cortical tracking of the acoustic envelope. Cortical envelope tracking was stronger while listening to atonal music, potentially reflecting listeners’ violated pitch expectations and increased attention allocation. Envelope tracking was also stronger with more expertise and enjoyment. Furthermore, we showed cortical tracking of pitch surprisal (using IDyOM), which suggests that listeners’ expectations match those computed by the IDyOM model, with higher surprisal for atonal music. Behaviorally, we measured participants’ ability to finger‐tap to the beat of tonal and atonal sequences in two experiments. Finger‐tapping performance was better in the tonal condition, indicating a positive effect of pitch predictability on behavioral rhythm processing. Cortical envelope tracking predicted tapping performance for tonal music, as did pitch‐surprisal tracking for atonal music, indicating that high and low predictability might impose different processing regimes. Taken together, our results show various ways that top‐down factors impact musical rhythm processing.

## Graphical abstract

This study compares rhythm processing under two conditions—high pitch predictability (tonal music) and low pitch predictability (atonal music)—while keeping the temporal information of the stimuli constant. More predictable pitch is associated with better behavioral rhythm tracking (finger‐tapping) and decreased cortical rhythm tracking, potentially indicating increased attentional focus. Musical expertise and enjoyment of the stimuli increase cortical tracking. Overall, top‐down factors show various influences on rhythm tracking.

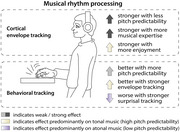

## INTRODUCTION

The cortical tracking of continuous auditory stimuli, such as music and speech, has been the topic of intense investigation in the past years.^1^, ^2^, ^3^ Cortical tracking usually refers to the neural signal matching slow amplitude fluctuations in the acoustic signal and is quantified by neural alignment to the stimulus envelope; it is thought to reflect the processing of the rhythmic structure.^4^, ^5^, ^6^ Although mostly investigated in speech, recent findings suggest that the processing of naturalistic music might rely on comparable mechanisms.^7^, ^8^, ^9^ Cortical tracking is influenced by numerous top‐down factors, but their interaction and relative importance are poorly understood. For example, increased attention and listening effort generally leads to stronger speech tracking.^10^, ^11^, ^12^, ^13^, ^14^ Conversely, both speech and music tracking are enhanced with language proficiency, music proficiency, and prior knowledge.^6^, ^8^, ^15^, ^16^, ^17^ Other factors, such as the influence of enjoyment on the cortical tracking of music, have also recently elicited researchers’ interest.^18^ Overall, any study of cortical tracking of rhythmic stimuli needs to take into account stimuli and listener characteristics, which is one driver of the present study.

Recent studies on the cortical tracking of music have shown that the auditory cortex tracks not only the acoustic envelope but also melodic expectations, modeled as surprisal values.^19^, ^20^, ^21^, ^22^, ^23^ These studies suggest that humans automatically process melodic expectations while listening to naturalistic, continuous stimuli.^24^ Here, we examine the cortical tracking of pitch surprisal using music stimuli with different levels of pitch predictability, namely, tonal and atonal music excerpts. Music that is composed according to (Western) tonal principles has an intrinsic hierarchical pitch organization.^25^ Therefore, this compositional style results in far more predictable pitch sequences than atonal music,^26^ which is based on the compositional principle that all 12 tones within an octave are equiprobable. The few studies that have been conducted using atonal music show that the resulting lack of a hierarchical pitch organization negatively affects memorization,^27^ recognition,^28^, ^29^, ^30^ and the strength of melodic expectations^31^ (for reviews, see Refs. 26, 32, 33). Electrophysiological research suggests that weaker expectancies in atonal music particularly affect later attention‐related processing stages.^34^, ^35^ Taken together, atonal music seems to present specific perceptual challenges to listeners, in particular related to melodic expectations.

In the context of musical rhythm perception, finger‐tapping is often used as a behavioral tracking measure to assess rhythm skills.^36^, ^37^, ^38^ The present study addresses the little‐known relationship between behavioral tracking (measured by finger‐tapping) and cortical envelope tracking (measured by electroencephalogram [EEG] recordings of listening participants) of musical rhythm in the context of varying pitch predictability. While temporal predictability has been shown to increase pitch discrimination performance,^39^, ^40^ it is currently unclear whether pitch predictability affects the ability to behaviorally track naturalistic musical rhythms.

Here, we investigated whether cortical tracking of the music envelope, usually an indicator of rhythm processing, is modulated by pitch surprisal in two continuous, naturalistic stimulus conditions: tonal (high pitch predictability) and atonal (low pitch predictability) music. In the main experiment, we analyzed participants’ EEG during passive listening, focusing on cortical envelope and surprisal tracking. We also investigated the role of enjoyment and musical expertise for cortical envelope tracking. In both the main and follow‐up replication experiments, we used a behavioral measure of rhythm perception (finger‐tapping) to analyze whether cortical tracking is behaviorally relevant and whether pitch predictability influences behavioral tracking. We expected that high pitch predictability in the tonal condition would be associated with better behavioral rhythm tracking than low pitch predictability in the atonal condition (see preregistration: https://osf.io/qctpj). Due to complex and opposing effects of attention and previous experience (for example, Ref. 41), we did not hypothesize a priori on whether cortical envelope tracking would be stronger in the tonal or atonal condition.

## MATERIALS AND METHODS

### Participants

Twenty volunteers participated in the main study (14 female, 6 male; 18−26 years old; *M * =  20.95, *SD * =  1.88). It was initially planned to test 24 participants (preregistration: https://osf.io/qctpj), but data collection had to be halted due to the COVID‐19 pandemic. However, the sample size analysis was based on a previous study^27^ (*d*  =  0.64, *α*  =  0.05, *β*  =  0.80; see preregistration) and yielded a desired sample size of *N * =  21, which was close to being achieved. In addition, we tested a further 52 participants in a behavioral follow‐up experiment (see below). Participants in the main study were right‐handed (*N* = 19) or ambidextrous (*N* = 1).^42^ Quick Hearing Check self‐reports^43^ indicated that 19 participants had normal hearing, while one reported a score that might suggest slightly diminished hearing (score of 27/60, hearing test recommended from score 20). All participants reported never having received a diagnosis of neurological/psychological disorders or dyslexia. Participants self‐assessed their musical expertise on a scale from 1 to 3 (“none,” “some,” “a great deal”; *M*  =  1.95, *SD*  =  0.76). Six participants reported no musical expertise. Most participants (*N*  =  18) were unfamiliar with the musical stimuli, and although two reported familiarity with the music, they could not name the piece nor composer.

The study was approved by the School of Social Sciences Research Ethics Committee at the University of Dundee (approval number: UoD‐SoSS‐PSY‐UG‐2019‐84) and adhered to the guidelines for the treatment of human participants in the Declaration of Helsinki. Participants were reimbursed for their time with £15. The main analyses were preregistered before starting data analysis (https://osf.io/qctpj). Some deviations from the preregistration occurred when processing the actual data, or due to new developments in data analysis, and are described where appropriate below.

### Musical stimuli

**FIGURE 1:**
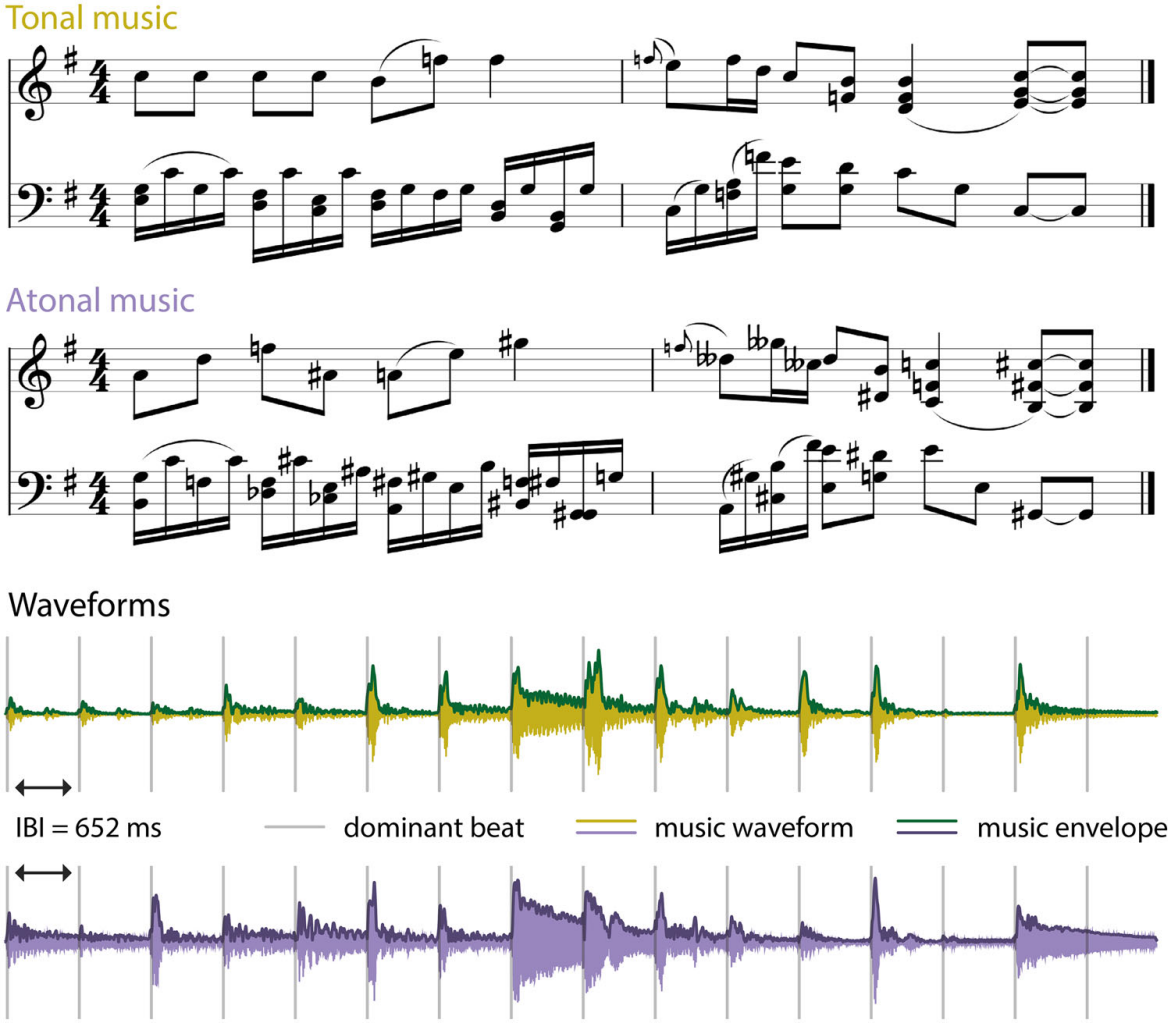
Examples of sheet music and waveforms. *Top*: Sheet music for two bars of the tonal condition (original music: “Mozart's Sonata No. 5 in G Major,” “K. 283: II. Andante”) and the same bars in the atonal/nonmelodic condition. *Bottom*: Waveforms of the same bars in the tonal (green) and atonal (purple) condition, including the music envelope. Gray bars represent the positions of the dominant beat with an inter‐beat interval of 652 ms (1.52 Hz).

**FIGURE 2:**
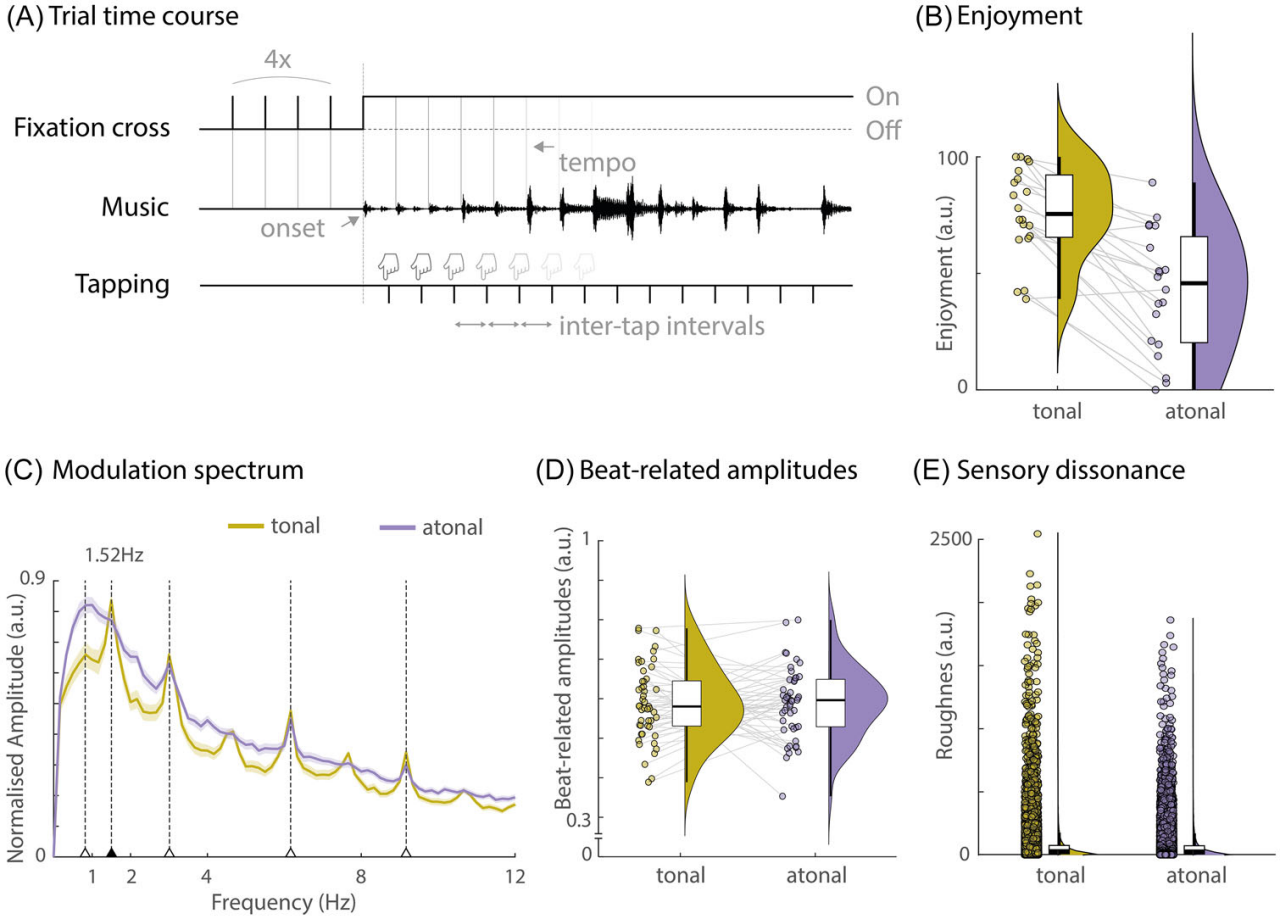
Behavioral paradigm and stimulus properties. (A) Depiction of the trial time course for the behavioral tracking task. Before the music started for each 2‐bar trial, the dominant beat frequency (1.52 Hz) was indicated visually by flashing a fixation cross four times at that frequency. Participants tapped their finger to the dominant beat of the music once the music started. (B) Enjoyment ratings for both 5‐min tonal and atonal excerpts by all participants in the main experiment (*N*  =  20). Overall, participants rated the tonal condition as more pleasant/enjoyable than the atonal/nonmelodic condition. (C) Modulation spectrum of both tonal and atonal 5‐min excerpts. Thick lines indicate average values across 6‐s segments, with shaded areas representing standard error of the mean. Beat/meter‐related frequencies are indicated by arrows and dotted lines. (D) Averaged amplitude values of beat‐related frequencies (as shown in C) for both tonal and atonal excerpts. (E) Sensory dissonance, assessed via roughness. There was a small difference in that the tonal condition showed larger roughness values than the atonal condition. *Note*: Distribution plots show individual data points, box plots (including median, interquartile ranges, and minimum/maximum), and kernel density estimates. Abbreviation: a.u., arbitrary units.

Tonal and atonal polyphonic piano stimuli were used (see Figure 1, top). For the tonal condition, we used an excerpt from W.A. Mozart's “Sonata No. 5 in G Major, K. 283.” The excerpt was taken from the second movement (“II. Andante”). The atonal piece was a manipulated version of this excerpt, created by randomly shifting the pitch of each note from one to nine semitones up or down the musical scale (using GuitarPro v7.5), corresponding to 100–900 cents. Therefore, notes no longer formed harmonic relationships, while the timing of each note remained the same (see control analysis of note onset surprisal in Supplementary Analyses and Figure S2). Our manipulation in the control condition shares some characteristics of atonal music, such as a lack of hierarchical pitch organization. However, it is important to note that this is not entirely comparable with atonal music created by composers and might be better characterized as *nonmelodic*. To ease reading, we opted to keep the overall label atonal for our control condition. Overall, the music in both conditions contained identical timbre, velocity, and rhythm. Each excerpt was approximately 5‐min long (292 s) and had a standard 4/4 time signature. The tempo of the pieces was 46 beats per minute (bpm), but because eighth note measures were consistently used, the dominant beat was 92 bpm (Figure 1). This equaled a rate of 1.52 Hz (see modulation spectrum in Figure 2), and the beats were 652 ms apart. For the finger‐tapping task, unique two‐bar segments from the same pieces were extracted per condition (18 segments, each 10.4 s long). All music pieces were presented at a sampling rate of 44,100 Hz. All stimuli are available on the OSF server (https://osf.io/3gf6k/).

### Procedure and task

Participants performed the EEG experiment in a quiet room. They sat comfortably, approximately 110 cm from a Benq computer screen (22.65 × 13.39 inches; 1920 × 1080 pixels resolution). On‐screen instructions were presented in black, size 30 Consolas font, and displayed against a gray background. Participants could adjust the volume of the sound to a comfortable level before the start of the experimental blocks. Musical stimuli were presented using E‐Prime 3.0 software (Psychology Software Tools Inc., 2016), and were listened to through high‐quality wired headphones (Creative, Draco HS880). Participants first passively listened to the 5‐min tonal and atonal music excerpts (randomized order). Participants started the music self‐paced. A 5‐s countdown was shown, before an X appeared at music onset, on which participants fixated throughout the music listening. After each music piece, participants rated how pleasant they had found the music. We used a Visual Analog Scale, on which participants could rate their enjoyment by drawing a vertical line between *Not pleasant* and *Very pleasant*.

After the passive listening blocks, participants performed a finger‐tapping task to measure behavioral rhythm tracking in the tonal and atonal conditions. Thirty‐six unique trials (18 per condition) were presented in four blocks (two tonal and two atonal) of nine trials each. The order of blocks and of trials within each block was randomized across participants. Each trial was started self‐paced and began with a visual presentation of the dominant beat (i.e., eighth notes). For this, an X flashed four times at the beat frequency before the music started (see Figure 2A). The dominant beat was presented visually and not acoustically, so as not to interfere with music processing. Participants then tapped with the index finger of their dominant hand on the outer “Enter” key of a silent keyboard to the dominant beat of the music. The length of the music segments (two bars each) required 16 finger taps per trial, resulting in approximately 288 taps per condition.

### Replication of behavioral results

To make sure that the behavioral effect found in the main experiment (more consistent finger‐tapping to tonal than atonal excerpts) was robust, we carried out a follow‐up replication study. All experimental procedures were approved by the Ethics Council of the Max Planck Society (Nr. 2017_12). The number of participants was *N* = 52 (33 female, 19 male), and their ages ranged between 20 and 41 years (*M*  =  26.6, *SD*  =  5.3 years). Most participants were right‐handed (*N*  =  43), some were left‐handed (*N*  =  6) or ambidextrous (*N*  =  3). The procedure was identical to the main experiment, with the exception that four bars were used for each trial, thus doubling the time for finger‐tapping per trial. This led to 10 unique tonal and 10 unique atonal trials, each 20.9 s long. Each trial required 32 finger taps, resulting in 320 taps per condition. Furthermore, all tonal and atonal trials were presented in random order (in contrast to tonal and atonal blocks as in the main experiment).

### Analysis of behavioral data

The inter‐tap intervals of participants’ keyboard taps for each trial were preprocessed in several ways to clean up the data. First, the first two finger taps (i.e., before 981 ms) at the beginning of the trial were excluded from further analysis to allow participants to hear two eighth notes to inform their tapping. Trials with fewer than 50% of expected remaining inter‐tap intervals were excluded (i.e., 6 necessary inter‐tap intervals in the original experiment and 15 in the replication experiment). Inter‐tap intervals of faster than 50 ms (indicating involuntary movements) and slower than 3000 ms (indicating idling) were removed. Within each participant and condition, trials with intervals of larger than three standard deviations from the mean were also excluded.^44^, ^45^, ^46^ At the participant level, our criterion was to exclude outlier data of larger than three standard deviations from the mean per condition (*N* = 0 in the original experiment, *N* = 0 in the replication). During the replication experiment, three participants misunderstood the instructions and consistently tapped to fast 16th notes. These three participants were excluded, resulting in 49 participants who were included in the final analyses. Finger‐tapping performance per trial, condition, and participant was quantified as the median absolute deviation (MAD) of the inter‐tap intervals per trial, a robust measure of dispersion^47^ that precisely captures the variability in tapping timing. As the MAD is based on median values, it is less affected by outliers than measures based on the mean, such as variance or the coefficient of variation.^48^ Enjoyment ratings on the visual analog scales for both the tonal and atonal excerpts were analyzed on a scale between 0 and 100, in increments of 1 (a.u., see Figure 2B). We also preregistered analyzing participants’ tapping accuracy (the synchronization between tap and beat) in addition to their variability (https://osf.io/qctpj). We were unable to carry out this analysis because we did not have information about the trigger‐sound latency of the used experimental setup. Data collection had to be stopped abruptly during the COVID‐19 pandemic, and access to the laboratory was restricted. After access was reinstated in 2021, computers and operating systems had been updated, and latency measurements were not possible for the original setup. However, while tapping variability is not a direct measure of tracking (or synchronization to the beat), it allows similar conclusions about rhythm performance (i.e., larger tapping variability indicates worse synchronization).

### EEG data acquisition and preprocessing

EEGs were recorded from 32 scalp electrodes, using a BioSemi ActiveTwo system (sampling rate 512 Hz). Electrodes were placed according to the International 10–20 system. Electrodes with an offset of greater/less than ±20 mV were adjusted. Ultimately, electrode offset was always below an absolute value of 30 mV before the experiment began. Horizontal eye‐movements were captured by two electro‐oculographic electrodes placed at the outer canthus of each eye. To capture vertical eye‐movements and blinks, a further two electrodes were positioned above and below the participants’ left eye.

Preprocessing of the EEG data was conducted using FieldTrip^49^ functions in MATLAB 2021a (MathWorks Inc.). For both 5‐min excerpts used during passive listening, we cut out epochs of 304 s (300 s stimulation time from music onset, plus an additional 2 s leading and trailing windows). Data were initially re‐referenced to *Cz* and bandpass filtered between 0.1 and 100 Hz (third‐order Butterworth filter, forward and reverse). Data were then visually inspected using summary metrics (maximum value and *z*‐value in each channel), and noisy channels were removed and interpolated using triangulation. A maximum of four channels was removed per participant (*M* = 2.47, *SD* = 0.91). Before independent component analysis was conducted to identify blinks and artifacts, data were re‐referenced to average reference (Bertrand et al. 1985).^50^ On average, *M* = 2.16 (*SD* = 0.69) components per participant were removed from the data.

### Music envelope preprocessing

To analyze the tracking of the music signal, we extracted the wideband music envelope. We first down‐sampled each music excerpt to a sampling rate of 150 Hz.^1^ Acoustic waveforms were then filtered into eight frequency bands (between 100 and 8000 Hz, third‐order Butterworth filter, forward and reverse) that were equidistant on the cochlear frequency map.^51^ The signal in each of these eight frequency bands was Hilbert‐transformed, and the magnitude extracted before they were averaged for the wideband music envelope, which was used for further analyses.

### Pitch surprisal modeling

Surprisal during music listening refers to how expected a certain musical event is. Some note sequences are extremely prevalent across Western classical music, thus creating high expectations and low surprisal for an audience listening to them. To provide a computational account of music surprisal in the stimuli used, we relied on a model that learns the statistical regularities of music.^52^ Based on a variable‐order Markov model, IDyOM Information Dynamics Of Music^53^, ^54^ simulates listeners’ expectations while listening to music by collecting statistical structures of note sequences over *n*‐orders on a training corpus set. Here, the training corpus was composed of a collection of Western folk songs (a subset of the Essen Folksong Collection containing 953 melodies), so as to accurately model surprisal for typical Western listeners.^21^, ^55^ Specifically, the long‐term component (LTM) of the model collects the sequence statistics over *n*‐orders of the training set, while the short‐term component (STM) dynamically collects the local context over *n*‐orders for each testing melody. For each note of the testing melodies, the model outputs a probability distribution of pitch obtained from merging the distributions obtained by the STM and the LTM (for more details, see Ref. 53). By comparing the pitch ground truth to the probability predicted by the model, a surprisal value is obtained. Formally, the surprisal (or information content) is the log‐negative to the base 2 of the probability of the note, measured in bits. It essentially represents the expectedness of each note given the STM (e.g., the local context) and LTM (e.g., the long‐term exposure to a musical style or culture). A surprisal value of three bits (as is the average for notes in the tonal melody) means the event is as surprising as resolving 2^3^ equally likely choices. If surprisal is high, prediction error is also high, and vice versa.^52^ The choice of IDyOM was motivated by numerous empirical evidence that it can accurately model a listener's internal representation of musical regularities, both using neural and behavioral data.^19^, ^21^, ^24^, ^56^, ^57^ Since IDyOM in its current development only takes monophonic MIDI inputs, we reduced the complete score of each excerpt into a monophonic version that contained the melody and the bass line. The pitch surprisal values for each note were then used to build a continuous signal, with surprisal values making up the amplitude for the duration of the respective note. This initial step function was smoothed by convoluting it with a Gaussian filter (*sigma* = 50). The continuous surprisal signal was created to have the same sampling rate as the EEG signal (150 Hz).

### Mutual information analysis

The correspondence between the continuous EEG signal and envelope and surprisal signals (i.e., cortical envelope tracking and cortical surprisal tracking) was analyzed using a Gaussian copula mutual information (MI) framework.^58^, ^59^ In this approach, which is optimized for neurophysiological data, Gaussian copulas are used to normalize the continuous, analytical signals.^58^ The first 500 ms of the signals were removed from analysis to avoid contamination with strong transient evoked responses at the start of the music. MI (in bits) between the EEG signal and the music envelope was computed with both signals filtered at the dominant beat frequency range (0.5–3 Hz). We opted to deviate from the preregistered fixed stimulus–brain lag of 100 ms, as using a participant‐specific optimal time lag has emerged as a robust approach for tracking analyses using MI and phase coherence.^8^, ^60^, ^61^ The optimal stimulus–brain lag was based on the individual phase coherence^8^ peak at auditory electrode *Cz*, averaged for slow frequencies between 1 and 12 Hz (before the narrow band‐pass filtering described above). Initial coherence values were computed for nine lags between 40 and 200 ms in steps of 20 ms. This was first done separately for the tonal and atonal conditions. Values were averaged across conditions before choosing the peak lag for each individual.

MI between the EEG signal and the pitch surprisal signals was computed with signals that were bandpass filtered between 0.1 and 50 Hz. As the analysis sampling rate was 150 Hz, filtering up to 50 Hz allowed for this frequency to be robustly reflected in the data (1/3 of the sampling frequency). This wide range was chosen as no clear assumptions about a specific, narrow‐band frequency range could be made and prediction processes have been shown across multiple frequency bands.^62^, ^63^ The surprisal‐tracking analysis included surprisal values for both the melody and bass lines jointly, capitalizing on the multivariate capabilities of the used MI approach.^58^ Apart from the wider frequency band, the analysis of surprisal tracking was equivalent to that of envelope tracking, including the same individual stimulus–brain lags.

Each MI value was computed per participant, condition, and electrode. The results of these analyses will be referred to as cortical (envelope or surprisal) tracking, and we do not make assumptions about the underlying mechanisms (e.g., cortical entrainment), as these are still debated.^64^, ^65^, ^66^

### Statistical analyses

To test the statistical significance of MI values for envelope and surprisal tracking against chance, we implemented a cluster‐based permutation approach.^67^ For this, 3000 permutations were computed per participant, condition, and electrode. Specifically, to create permuted data, we segmented the continuous envelope/surprisal signals into 1 s segments and shuffled the segments randomly. This kept the statistical properties of the signal but destroyed the temporal relationship between the music and brain signals. MI was then computed between the brain signal and the 3000 shuffled envelope/surprisal signals. The group level mean was then tested against the 95th percentile of the random group mean distribution of the 3000 permutations, essentially implementing a one‐sided randomization test at *p* < 0.05.^60^ Only clusters with a minimum of two electrodes, and where MI values exceeded the critical value as defined by the 95% percentile of the permutation distribution, were selected for cluster‐level statistics. For observed clusters, the largest cluster‐level statistic (here, *t*‐values) from each permutation were aggregated into a null distribution, and clusters were considered statistically significant if they exceeded the 95th percentile of the null distribution. This controlled for the family‐wise error rate at the cluster level.

For the comparison between the two conditions, *t*‐values were computed using the real MI values, as well as the 3000 MI values from the shuffled data. These real and permuted data were then compared, again using a cluster‐based permutation test, with a critical *t‐*value of 2.1, which represents the critical value of the Student's *t* distribution for 20 participants and a two‐tailed probability of *p* = 0.05.^1^ As above, clusters had to consist of a minimum of two electrodes to be considered for cluster‐level statistics.

Pearson's correlations between cortical tracking and behavioral measures (tapping variability, musical competency, and enjoyment) were computed between the behavioral measures and the true MI values, as well as between the behavioral measures and the 3000 permuted MI values. Before comparing the true *r* values with the permutation distribution using cluster‐based permutation (using the same minimum cluster size and tested against the 95% percentile of the permutation distribution, as above), Pearson's *r* values were transformed to be normally distributed using Fisher's *z*‐transformation (for example, see Ref. 68). For all cluster‐based permutation analyses, initial clusters were chosen at an alpha level of *p* < 0.05. As an indicator of effect sizes, we either report Cohen's *d* for peak electrodes in the case of *t* or *r* values,^69^, ^70^ or summed MI values within each significant cluster (*MI*_sum_).^1^

To be able to draw evidence‐based conclusions about the laterality of our results, we explicitly tested for hemispheric lateralization.^71^, ^72^ Hemispheric differences in cortical tracking have been theoretically assumed and occasionally been found experimentally for the music envelope.^6^, ^73^ The participant‐specific results (e.g., MI values) were extracted for significant electrodes in one hemisphere and in corresponding contralateral electrodes. We then averaged these values within each hemisphere and calculated the between‐hemispheres differences with a group‐level Student's *t*‐test (two‐sided). *p*‐Values were corrected for multiple comparisons using false detection rate (FDR) correction at the level of 5%.^74^

All tests were two‐tailed, except for the comparison of finger‐tapping variability between the tonal and atonal conditions. These comparisons were one‐tailed, as we had an *a priori*‐directed hypothesis that finger‐tapping in the atonal condition would be more variable than in the tonal condition (see preregistration, https://osf.io/qctpj). All statistical analyses described above were implemented in MATLAB 2021a (MathWorks Inc.).

## RESULTS

### Differences between tonal and atonal music stimuli

**FIGURE 3:**
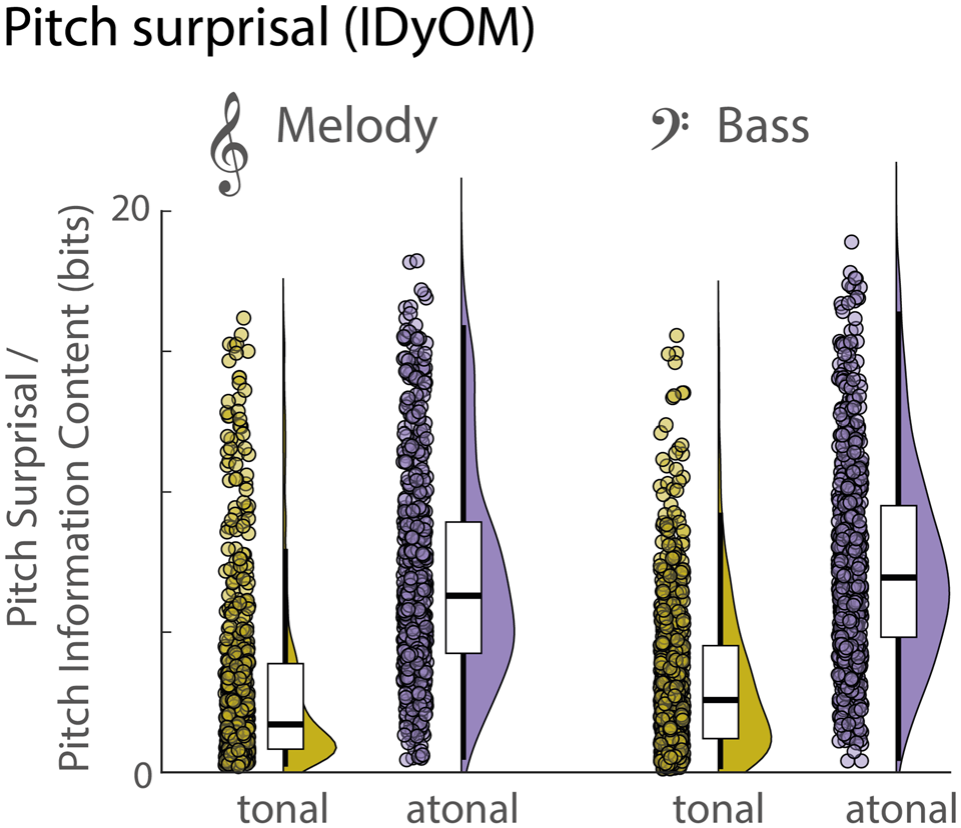
Pitch surprisal (quantified through pitch information content) values for each note in the melody and bass lines of both tonal and atonal 5‐min excerpts. Points indicate data for all notes, violin plots show kernel density estimates, and boxplots show median interquartile ranges, and minimum/maximum. Pitch surprisal was higher for the atonal than the tonal condition.

As intended, the pitch surprisal was overall higher for the atonal than the tonal stimuli (see Figure 3). Pitch surprisal (or pitch information content) was computed for all notes and separately for melody and bass lines using IDyOM.^53^ The surprisal was estimated by comparing the pitch ground truth of a note to its predicted value in the model's output distribution. For the melody line, pitch surprisal was on average *M* = 3.02 bits (*SD* = 3.28 bits) in the tonal condition, and *M* = 6.96 bits (*SD* = 3.78 bits) in the atonal condition (see Figure 3). A Student's *t*‐test confirmed that surprisal values were statistically larger in the atonal than the tonal condition (*t*(597) = −25.21, *p* < 0.001, Cohen's *d* = −1.03). Likewise, pitch surprisal in the bass line was higher in the atonal than in the tonal condition (tonal: *M* = 3.26 bits, *SD* = 2.72 bits; atonal: *M* = 7.38 bits, *SD* = 3.54 bits; *t*(722) = −28.41, *p* < 0.001, Cohen's *d*  =  −1.06).

Furthermore, participants were asked to rate how pleasant they found listening to the tonal and atonal music stimuli (on a scale effectively analyzed from 0 to 100 a.u., Figure 2B). The enjoyment ratings indicated that participants found the tonal excerpt more pleasant than the atonal excerpt (tonal: *M*  =  76.18, *SD*  =  19.31; atonal: *M*  =  42.53, *SD*  =  25.99; *t*(19)  =  6.47, *p* < 0.001, Cohen's *d*  =  1.45).

We also computed the modulation spectrum^74^ for both conditions between 0 and 12 Hz (Figure 2C). For this, the 5‐min excerpts were segmented into 6‐s chunks.^75^ This showed several peaks at beat‐related frequencies (i.e., subharmonic and harmonics of 1.52 Hz). A comparison of average beat‐related frequencies (see Refs. 76, 77 for excerpts), across both conditions showed no statistical difference (tonal: *M*  =  0.589, *SD*  =  0.093; atonal: *M*  =  0.590, *SD*  =  0.088; *t*(17)  =  −0.06, *p*  =  0.954, Cohen's *d*  =  0.008; Figure 2D). This indicates that the amplitudes of beat‐related peaks in the modulation spectrum were comparable in both conditions.

To assess whether the atonal condition shows any drastic perceptive pitch differences compared with the tonal condition, we computed roughness as a measure of sensory dissonance using the MIR toolbox.^78^ The default frame length of 50 ms (25 ms overlap) was used. The roughness in the tonal condition was slightly higher than in the atonal condition (tonal: *M*  =  82.85, *SD*  =  155.73; atonal: *M*  =  60.18, *SD*  =  122.02; *t*(11,674)  =  15.25, *p* < 0.001, Cohen's *d*  =  0.14; Figure 2E). The effect was very small (according to Cohen's *d* conventions) but implies at least that the atonal condition did not show more sensory dissonance than the tonal condition.

### Behavioral tracking: Finger‐tapping is more variable in the atonal than the tonal condition

**FIGURE 4:**
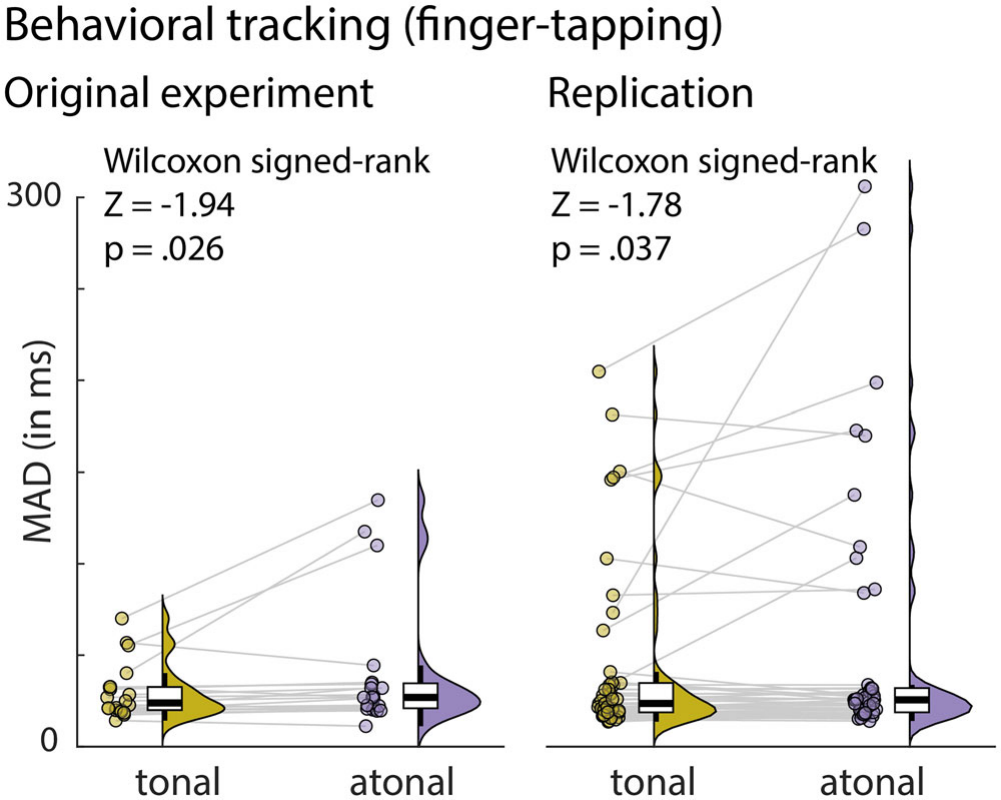
Behavioral tracking results of the main experiment (*N*  =  20) and the replication experiment (*N*  =  49). Shown is the median absolute deviation (MAD) in ms for inter‐tap intervals in both the tonal and atonal/nonmelodic condition. Points indicate individual data for all participants, violin plots show kernel density estimates, and boxplots show median interquartile ranges and minimum/maximum.

To test how pitch predictability influences behavioral rhythm tracking, we first analyzed differences in inter‐tap intervals between the tonal and atonal conditions. To mitigate the effect of potential outliers on the group level (see Figure 4), we used a nonparametric approach. In Experiment 1, the MAD of inter‐tap intervals in the tonal condition was on average *M*  =  29.72 ms (*SD*  =  14.99 ms). In the atonal condition, the MAD was slightly higher, on average *M*  =  40.30 ms (*SD*  =  35.66 ms). A direct comparison using a Wilcoxon signed rank test indicated that tapping performance was significantly more variable in the atonal than the tonal condition (difference  =  10.6 ms, *Z*  =  −1.94, *p*  =  0.026, *one‐tailed*). Out of the 20 participants, 15 (75%) had more variable inter‐tap intervals when tapping in the atonal than in the tonal condition.

We performed the same analysis for the behavioral follow‐up study, which had more than twice as many participants, and in which individual trials were twice as long as in the original experiment. The MAD of inter‐tap intervals in the tonal condition was on average *M*  =  42.56 ms (*SD*  =  46.24 ms). In the atonal condition, the MAD was again slightly higher, on average *M*  =  52.42 ms (*SD*  =  67.33 ms). A Wilcoxon signed rank test confirmed that tapping performance was significantly more variable in the atonal than the tonal condition (difference  =  9.9 ms, *Z*  =  −1.78, *p*  =  0.037, *one‐tailed*). Out of the 49 participants, 30 (61.2%) had more variable inter‐tap intervals when tapping in the atonal than in the tonal condition. Together, the results of the original and replication experiments indicate that there is a small but replicable effect of tonality on finger‐tapping variability: When listening to tonal music, participants tap to the beat more consistently than when listening to atonal music.

### Cortical tracking of the music envelope

**FIGURE 5:**
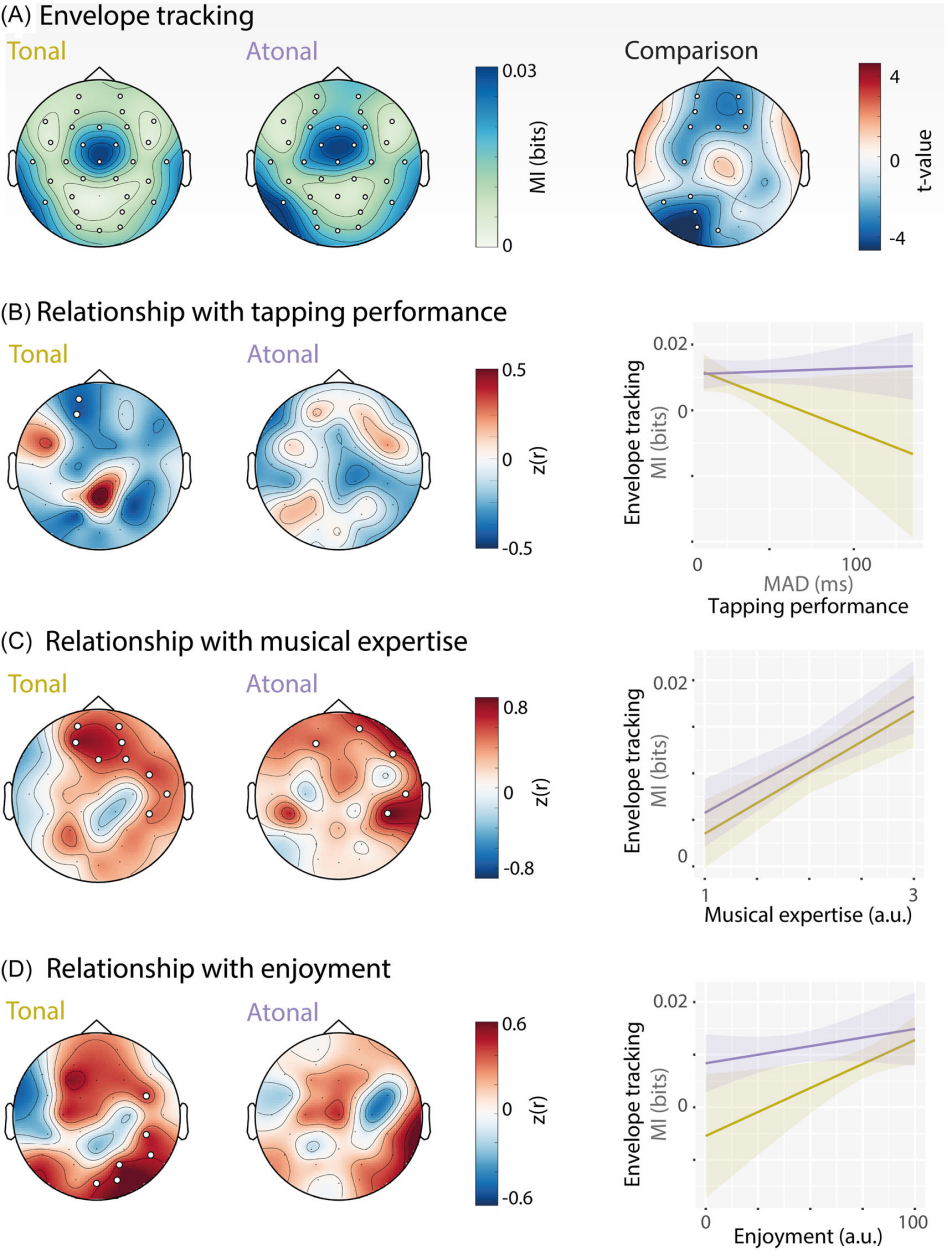
Cortical tracking of acoustic music envelope and its relationship with behavioral measures. (A) Topography of cortical envelope tracking assessed through mutual information (in bits) for both conditions. The right topography shows *t*‐values from a direct comparison between tonal and atonal/nonmelodic music. (B) Correlation between cortical envelope tracking and participants’ finger‐tapping performance. Envelope tracking predicted finger‐tapping performance only in the tonal condition, in a left‐frontal cluster. Here, stronger envelope tracking was associated with better performance (i.e., less tapping variability). (C) Correlation between cortical envelope tracking and participants’ self‐reported musical expertise. Expertise predicted envelope tracking in both conditions, in a fronto‐right‐lateral cluster. Stronger envelope tracking was associated with more musical expertise. (D) Correlation between cortical envelope tracking and enjoyment. Enjoyment predicted envelope tracking in the tonal condition in fronto‐central and posterior electrodes. A regression model showed a significant main effect of enjoyment with no significant interaction. Perceiving the music as more pleasant was associated with stronger envelope tracking. *Note*: Significant electrodes are highlighted with white circles.

We first analyzed whether participants tracked the acoustic music envelope—band‐pass filtered around the dominant beat frequency—in the tonal and atonal conditions compared with chance level using cluster‐based permutation (Figure 5A). In the tonal condition, we found a large positive cluster of 31 electrodes that significantly tracked amplitude fluctuations (*p* < 0.001, *MI*_sum_  =  0.337). Equivalently, in the atonal condition, there was a positive cluster of 32 electrodes that showed significant envelope tracking (*p* < 0.001, *MI*_sum_  =  0.438). There was no evidence for hemispheric lateralization, neither in the tonal nor in the atonal condition (both *p*_FDR_ > 0.96). We then directly compared envelope tracking in both conditions. This resulted in one large fronto‐parietal negative cluster (*p* < 0.001, Cohen's *d*_peak_  =  −2.08, 13 electrodes). The negative cluster indicated that the acoustic music envelope was tracked more strongly in the atonal than the tonal condition. We confirmed these results in an additional conditional MI analysis, in which we partialled out the effect of pitch intervals on envelope tracking (see Supplemental Analyses and Figure S1).

### Envelope tracking for tonal music during passive listening predicts finger‐tapping performance

To test whether envelope tracking during passive listening predicted participants’ behavioral tracking of the beat (i.e., finger‐tapping performance), we correlated the MI values per electrode with participants’ average tapping variation across trials (MAD, Figure 5B), separately for each condition. We found one negative cluster over left‐frontal electrodes that predicted tapping variance in the tonal condition (*p*  =  0.042, Cohen's *d*_peak_  =  −0.95, 2 electrodes). This indicates that participants who showed stronger envelope tracking to tonal music had smaller variance (i.e., better performance) when tapping in the tonal condition compared to participants who had weaker envelope tracking. Envelope tracking when listening to atonal music did not significantly predict tapping performance in the atonal condition. To compare this relationship directly between the tonal and atonal conditions, we entered the average MI values of electrodes in the negative cluster (Figure 5B, left) as a predictor into a regression model (using RStudio 2024.04.2 and R 4.4.1), with *tonality* as an additional predictor, and an *envelope tracking × tonality* interaction term, and *finger‐tapping variability* (MAD) as an outcome variable. This overall model was not significant (*F*(3,36)  =  0.89, *p*  =  0.458) and only explained 6.88% of the variance. The model yielded no main effects (both *p* > 0.32) nor interaction (*p* > 0.30), which suggests that the effect of envelope tracking on finger‐tapping is small in the tonal condition, and not statistically different between the tonal and atonal conditions. Furthermore, when repeating the analysis with nonparametric Spearman's correlations instead of Pearson's correlations (to account for the observation that there are participants who show very variable tapping in both conditions; Figure 3), the pattern is similar, but the two‐electrode cluster in the tonal condition does not survive corrections for multiple comparisons. This means the effect of envelope tracking on finger‐tapping in the tonal condition is partly driven by individuals with poor tapping performance to tonal music.

### Expertise predicts envelope tracking for tonal and atonal music

Several previous studies have found that musical expertise is associated with stronger neural synchronization to music.^6^, ^8^, ^79^ We, therefore, tested the relationship between participants’ musical expertise and acoustic envelope tracking (Figure 5C). A large fronto‐temporal cluster showed a significant positive correlation between self‐assessed musical competency and music tracking in the tonal condition (*p*  =  0.001, Cohen's *d_peak_*  = 2.47, 9 electrodes). In the atonal condition, envelope tracking was also positively predicted by a fronto‐temporal cluster (*p*  =  0.002, Cohen's *d_peak_*  = 3.93, 5 electrodes). A regression model predicting *envelope tracking* (averaged across the electrodes included in the significant clusters reported above) from *tonality, musical expertise*, and their interaction (*F*(3,36) = 12.90, *p* < 0.001) explained 51.8% of the variance. Only the main effect of *expertise* was significant (*t*  =  4.43, *p* < 0.001; main effect of *tonality* and the interaction both *p* > 0.56). These results indicate that musical expertise is associated with enhanced tracking of the music envelope for both highly predictable (tonal) and minimally predictable (atonal) music but is unlikely to explain differences in tracking between conditions.

### Enjoyment predicts envelope tracking for tonal music

Participants also indicated how pleasant they found listening to the music after each condition using visual analog scales. These enjoyment ratings were significantly higher for tonal than atonal music (Cohen's *d*  =  1.45; see above and Figure 2B). Ratings were correlated with cortical envelope tracking across participants (Figure 5D). In the tonal condition, a right fronto‐parietal cluster showed a positive correlation between enjoyment and envelope tracking (*p*  =  0.002, Cohen's *d_peak _* =  1.43, 6 electrodes). No significant clusters emerged in the atonal condition. A regression model predicting *envelope tracking* (averaged across the electrodes included in the significant cluster reported above) from *enjoyment* ratings, *tonality*, and their interaction (*F*(3,36)  =  3.15, *p*  =  0.037) explained 20.8% of the variance. The main effects of *enjoyment* (*t*  =  2.48, *p*  =  0.018), and *tonality* (*t*  =  2.48, *p*  =  0.037) were significant, with no significant interaction (*t*  =  −1.28, *p*  =  0.210). This indicates that cortical tracking was increased with more *enjoyment* and this pattern was similar across conditions. It also indicates that cortical tracking was stronger in the atonal condition (mirroring the main effect between conditions seen in Figure 5A).

### Cortical tracking of pitch surprisal

**FIGURE 6:**
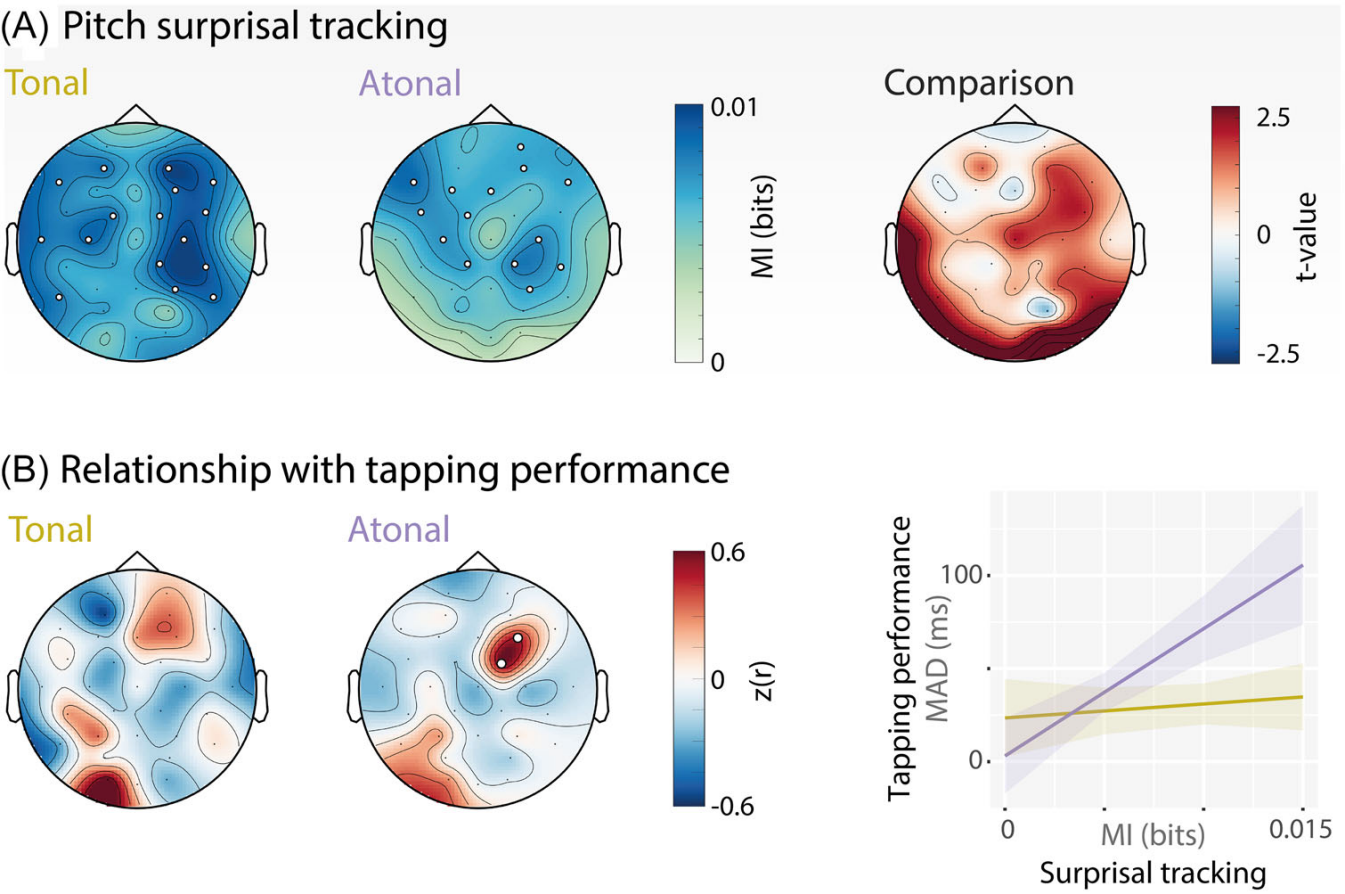
Cortical tracking of pitch surprisal performance. (A) Topography of cortical surprisal tracking assessed through mutual information (in bits) for both conditions. The right topography shows *t*‐values from a direct comparison between tonal and atonal/nonmelodic music. (B) Correlation between pitch surprisal tracking and participants’ finger‐tapping performance. Surprisal tracking predicted finger‐tapping performance only in the atonal condition, in a right‐frontal cluster. Here, stronger surprisal tracking was associated with worse performance (i.e., higher tapping variability). *Note*: Significant electrodes are highlighted with white circles.

Pitch surprisal was analyzed using the IDyOM model^53^ in both conditions. As expected, surprisal was higher for notes in the atonal than the tonal condition for both the melody and bass lines (Cohen's *d_peak_* =  −1.03 and Cohen's *d_peak_*  =  −1.06, respectively; see above and Figure 3). We first analyzed whether pitch surprisal was tracked in both conditions above chance level, using a multivariate analysis including surprisal in melody and bass lines (Figure 6A). In the tonal condition, pitch surprisal was tracked in a large bilateral cluster (*p* < 0.001, *MI*_sum_  =  0.073; 16 electrodes). Likewise, in the atonal condition, pitch surprisal was tracked in a bilateral electrode cluster (*p* < 0.001, *MI*_sum_  =  0.051; 14 electrodes). Although pitch tracking in the tonal condition appeared to be greater than in the atonal condition, directly comparing the tracking of pitch surprisal between both conditions yielded no statistically significant clusters. Likewise, although pitch surprisal tracking appeared larger in the right hemisphere, contrasting left‐ and right‐hemispheric cluster electrodes yielded no systematic lateralization of cortical tracking (tonal: *p*_FDR_ = 0.27 and atonal: *p*_FDR_ = 0.13, respectively). These results suggest that listeners form pitch expectations (i.e., prediction errors) comparable with the IDyOM model and that pitch surprisal is represented in the brain to a similar extent in tonal and atonal conditions.

### Surprisal tracking predicts finger‐tapping performance in the atonal condition

We also analyzed whether the extent to which participants tracked pitch surprisal predicted their finger‐tapping performance. This correlation analysis yielded no significant clusters in the tonal condition. However, in the atonal condition, the tracking of pitch surprisal was positively correlated with finger‐tapping performance in one frontocentral cluster (Figure 6B; *p*  =  0.027, Cohen's *d_peak_*  = 1.11, 2 electrodes). Again, to be able to draw conclusions about differences between the tonal and atonal conditions, we entered the average MI values of the positive cluster as a predictor into a regression model, with *tonality* as an additional predictor, a *surprisal tracking × tonality* interaction term, and *finger‐tapping variability* (MAD) as the outcome variable. The overall regression model (*F*(3,36)  =  7.26, *p* < 0.001) explained 37.6% of variance in finger‐tapping variability. Neither the main effect of *surprisal tracking* (*p* > 0.49) nor the main effect of *condition* (*p* > 0.16) reached significance. However, the interaction *surprisal tracking × condition* was statistically significant (*t*  =  3.20, *p* < 0.003). This interaction stemmed from an effect, exclusive to the atonal condition, where participants who tracked the pitch surprisal well finger‐tapped with higher variability than participants with relatively poor cortical tracking.

### Relationship between envelope tracking and surprisal tracking

**FIGURE 7:**
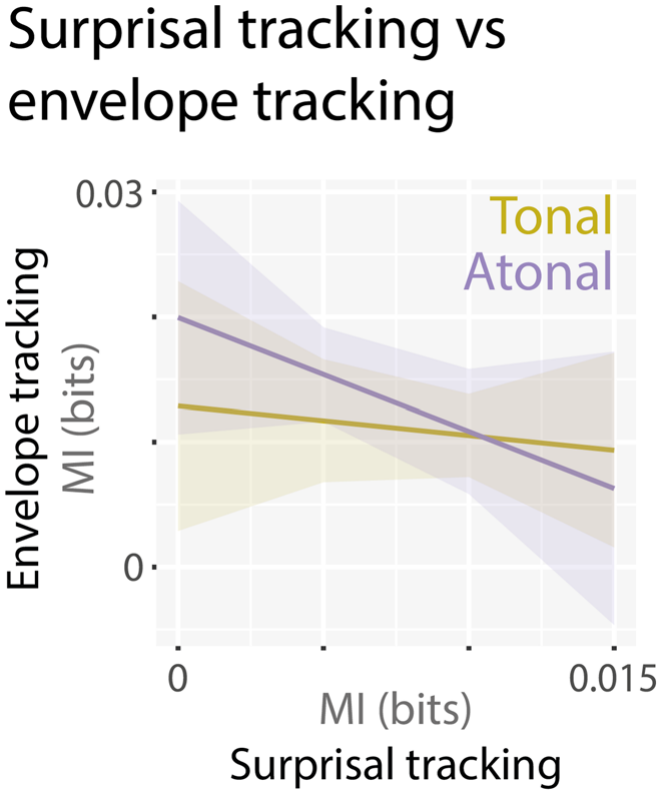
Relationship between cortical tracking of acoustic envelope and pitch surprisal.

Last, we were interested in the relationship between acoustic envelope tracking and pitch surprisal tracking because these measures have not previously been looked at together. We used the average MI value per cluster (as seen in Figures 5A and 6A) and participant in a regression model with *acoustic envelope tracking* as the outcome variable and *surprisal tracking*, *tonality*, and a *surprisal tracking × tonality* interaction term as predictors. The overall regression model was not significant (*F*(3,36)  =  1.40, *p*  =  0.258) and explained 10.5% of the variance. No main or interaction effect reached significance (all *p*‐values > 0.301; Figure 7). Thus, there seems to be no systematic relationship between acoustic envelope tracking and pitch surprisal tracking.

## DISCUSSION

**FIGURE 8:**
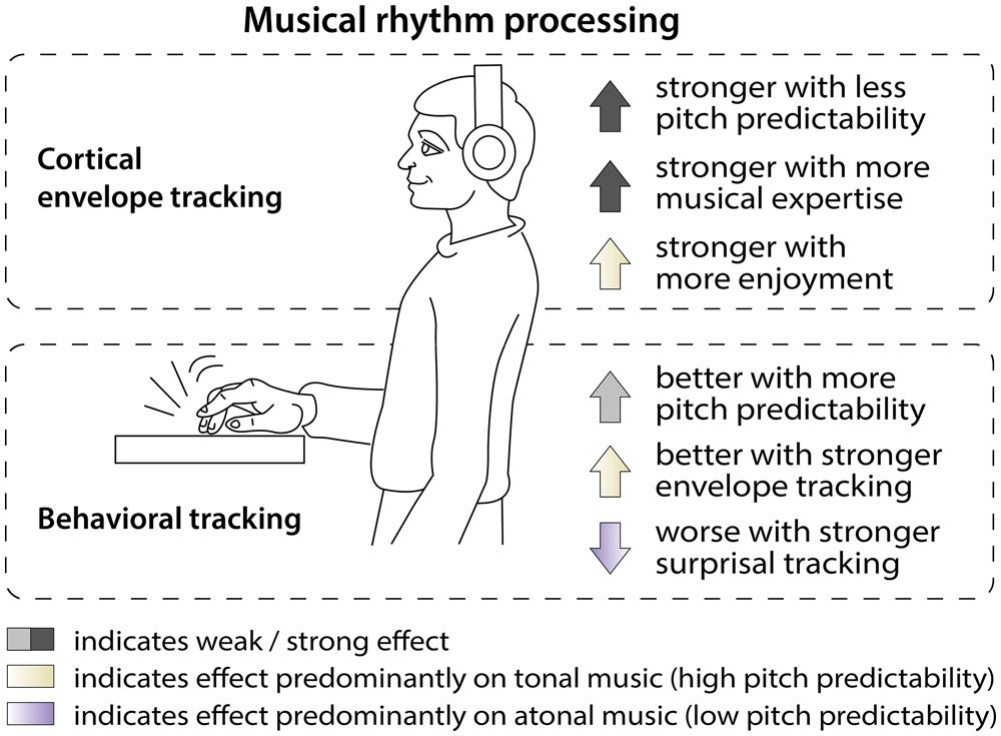
Overview of results.

In the present study, we show that the cortical representation of naturalistic continuous music, as measured through envelope tracking, reflects not only rhythm processing, but is also modulated by pitch predictability, musical expertise, and enjoyment (Figure 8). It is of note that our sample size in the main experiment (*N*  =  20) falls short of the preregistered and desired sample size, which was a consequence of the COVID‐19 pandemic.

### Pitch predictability affects rhythm processing as reflected in behavioral tracking

Atonal music can be used to study predictive processing under high‐uncertainty contexts.^26^, ^32^ While temporal predictability has been shown to increase pitch discrimination performance,^39^, ^40^ it is unclear whether long‐term pitch predictability affects the ability to behaviorally follow the beat, particularly in naturalistic musical stimuli. Crucially, in our main experiment and a replication study, we show that when listening to naturalistic music, pitch predictability (modeled on long‐term statistics, which reflect exposure to a musical culture, and short‐term melodic context) modulates the variability of finger‐tapping to the beat. In the tonal condition with higher pitch predictability, the finger‐tapping performance was more consistent (less variable inter‐tap interval) compared with the atonal low predictability condition. However, although the difference in tapping variability between tonal and atonal (i.e., nonmelodic) music could be replicated in a second sample, the effect was small in both experiments. Especially, in the replication experiment, only 61.2% of participants showed more variable tapping in the atonal condition (compared with 75% in the main experiment). The atonal music in our study contained a note timing structure that was identical to the tonal condition, but had generally lower pitch predictability, suggesting that this finding reflects a modulation of behavioral rhythm processing (i.e., finger‐tapping to the beat) by pitch predictability. This is in line with and extends previous studies showing expectation effects on musical perception.^39^, ^40^, ^54^, ^80^ An alternative (but not mutually exclusive) explanation is that perceived pleasantness modulated tapping variance. This is difficult to assess in the current study as pleasantness was tightly associated with the tonality of the music, but it could be manipulated independently in future studies. The current dataset also allows for analyses that test the influence of musical expertise on finger‐tapping or enjoyment, which were not carried out to keep this report concise but can be carried out with the publicly available data from the currrent study (https://osf.io/3gf6k/).

### Cortical tracking of the music envelope in tonal and atonal music

At the neural level, the music envelope was tracked in our study for both tonal and atonal music (Figure 5A). The tracking was observed in both conditions with a centro‐temporal topography in accordance with previous reports suggesting auditory cortex generators of the envelope tracking in speech^4^, ^5^ and music.^6^, ^23^, ^79^ Some studies reported a right lateralization for music envelope tracking^6^ in line with the asymmetric sampling in time theory.^81^, ^82^ The heterogeneous findings in the literature regarding whether hemispheric lateralization is observed have been related to various top‐down influences.^83^, ^84^, ^85^

Atonal music was more strongly tracked at frontal and left parietal electrodes compared with tonal music. Importantly, this was the case even though both conditions had an identical note timing structure and there were no significant acoustic differences in the modulation spectrum (Figure 2C,D). We interpret this as evidence that pitch predictability influences neural rhythm tracking. Our control analysis showed that even when the tracking of pitch intervals was partialled out, atonal music was still tracked stronger than tonal music (Figure S1). Furthermore, the atonal condition did not exhibit higher sensory dissonance. These control analyses support the interpretation that it is pitch predictability, and the not low‐level pitch differences that affect rhythm tracking. Our results from the neural data are also in line with our behavioral findings in that they suggest an effect of pitch predictability on rhythm processing. Interestingly, Weineck et al.^18^ speculated that more predictable music produces stronger neural synchronization, which our results contradict. However, their paradigm did not manipulate pitch predictability and results are, therefore, not directly comparable. A predictive coding approach^86^, ^87^ could provide a potential explanation for the observed effect. In the atonal condition, notes were generally less predictable than in the tonal condition. In line with the assumption of expectation suppression,^88^ this likely led to stronger neural prediction errors, which in turn might have resulted in stronger neural responses to the acoustic envelope,^89^ not unlike a mismatch‐negativity response (for example, Ref. 90). Accordingly, Kern et al.^21^ showed that surprising notes elicit stronger neural responses compared to predictable ones (see also Ref. 19).

A potential mechanism that might mediate the stronger neural response in the atonal condition is the shifting of attention (for example, Ref. 91). When stimuli are expected and prediction errors are small (as tends to be the case in our tonal condition), the brain can afford to spend little attentional and metabolic resources on those. In contrast, when stimuli are unexpected, and prediction errors are large (as tends to be the case in our atonal condition), this serves as an alerting response, and attention is allocated to the unexpected stimuli. In addition, it might have been more demanding to listen to the atonal stimulus. In the speech domain, increased attention as well as listening effort has long been associated with stronger neural tracking.^10^, ^11^, ^12^, ^13^, ^14^

### Increased envelope tracking in tonal music is related to better tapping performance

In the tonal condition, the cortical tracking of the musical envelope correlated with the behavioral tracking, with higher cortical tracking being associated with an increased ability to behaviorally follow the beat. This effect was small overall and seemed to be driven particularly by individuals with poor tapping performance (large tapping variability). The finding is in line with previous research showing a positive correlation between behavioral performance and cortical tracking of speech^92^, ^93^ and music.^6^ The relationship between cortical tracking and behavioral performance, however, might be more complex than this, as suggested for speech.^94^, ^95^ No correlation was observed in the atonal condition and furthermore, when the correlation effects were tested in a regression model that included both conditions and selected electrodes, no significant interaction effect was observed. This makes any interpretation regarding differences between tonal and atonal music difficult. A potential explanation is that under conditions of low pitch predictability (as in the atonal condition), the positive relationship between envelope tracking and behavioral tapping performance is confounded, perhaps due to the increased difficulty of trying to (unsuccessfully) make predictions about the upcoming notes, or due to increased allocation of attention. In summary, increased behavioral tracking was related to increased cortical tracking only in the tonal condition, albeit the effect was small.

### Pitch surprisal is cortically tracked in tonal and atonal music

We designed our stimuli so that the pitch predictability of the musical pieces was decreased in the atonal compared to the tonal condition (Figure 3). Importantly, our human listeners significantly tracked pitch surprisal in both conditions, which indicates that their pitch expectations (or their prediction errors) were comparable with the IDyOM model. Furthermore, there were no significant condition differences in the cortical tracking of pitch surprisal (Figure 6A). This suggests that participants’ neural models of pitch surprisal match the IDyOM computations, and the notes in the atonal condition elicited not only more surprisal in the IDyOM model but also in listeners’ neural response. Cortical tracking of pitch surprisal in naturalistic music has been rarely investigated. Two recent studies report melodic surprisal tracking in tonal music that was localized to bilateral superior temporal and Heschl's gyri (among others), and additionally showed either a central topography using EEG/EcoG,^19^ or a broad fronto‐temporal (and central) topography using magnetoencephalography.^21^ Overall, we found relatively widespread fronto‐temporal tracking of pitch surprisal across conditions, which is in line with the above results. Here, we show that listeners consistently track pitch surprisal not only for highly predictable music, as previously shown, but also for minimally predictable music.

### In atonal music lower pitch surprisal tracking is related to better tapping performance

Interestingly, the surprisal tracking strength was only correlated with the behavioral rhythm tracking performance in the atonal but not the tonal condition (as shown by the significant interaction between condition and surprisal tracking). Participants who tracked the pitch surprisal stronger, meaning they matched the high surprisal values from the IDyOM model, also showed more variability (worse performance) in their tapping in the atonal condition. Prediction tendencies have been suggested to vary across participants.^92^ Our measure of surprisal tracking might reflect such a tendency, with some individuals being more or less prone (or able) to make predictions. In the atonal condition with its high uncertainty, pitch predictions might be less informative for rhythm processing, and listeners who tend to make (stronger) predictions, which lead to high prediction errors, could have fewer resources to track the envelope and to perform well in the tapping task. Additionally, differences in how attention is allocated in the tonal and atonal conditions could play a role in the interaction between surprisal tracking and tapping performance. The few behavioral studies that looked at long‐term pitch surprisal tracking have not related it to the rhythm processing performance (for example, Ref. 21). Our results indicate that the negative effect of pitch surprisal tracking on behavioral rhythm processing might only be expected when pitch predictability is low and prediction errors are high, as in the case of atonal music. The cortical tracking of pitch surprisal was not systematically related to the cortical tracking of the acoustic envelope, at least not in our sample of 20 participants who underwent the EEG part of the study.

### Enjoyment and musical expertise are related to cortical envelope tracking

As expected, based on the literature,^26^, ^32^ the atonal music condition was rated as less pleasant than the tonal music condition. The individually perceived pleasure or enjoyment has a strong influence on our everyday music listening behavior (see Ref. 57). In the tonal condition, the enjoyment ratings correlated with the strength of cortical music envelope tracking, and this pattern was not statistically different in the atonal condition. The causal nature of this relationship remains unclear. Do listeners show stronger acoustic envelope tracking because they find the music more enjoyable, or do they find it more enjoyable because they have better acoustic envelope tracking? Interestingly, a previous study that investigated whether enjoyment influences neural synchronization to music did not find a significant effect.^18^ The discrepancy with our findings might be due to differences in experimental paradigms, music choices, quantification of music tracking, and analytical methods. If future studies replicate our findings, this suggests that the individual preference of listeners should be taken into account when measuring envelope tracking.

Additionally, musical expertise was correlated positively with the cortical envelope tracking of the music pieces at a cluster of frontal and right temporal electrodes for both the tonal and atonal conditions, with more expertise being related to stronger tracking. Our finding is in line with previous reports of the effects of musical expertise on cortical envelope tracking^6^, ^8^, ^79^ (but see Ref. 18 for a null effect). Here, we extend these previous results by showing that stronger envelope tracking by individuals with more musical expertise also extends to atonal music with low‐pitch predictability. The effect of musical expertise on the auditory processing of music has been related to increased auditory–motor coupling after musical training.^96^, ^97^

Another, not mutually exclusive, interpretation for the effects of both enjoyment and expertise could be that both are associated with generally more attention to the musical stimuli. That is, more musical expertise, as well as more enjoyment of the music, could result in unspecific increases of attention, which in turn could increase the signal‐to‐noise ratio of acoustic envelope tracking (for example, Refs. 11, 12, 98). To disentangle contributions of attention and other variables, future studies could implement measures of attention or listening effort into their paradigms.

## CONCLUSION

Our findings suggest that tracking of the envelope of naturalistic music, commonly associated with rhythm processing, is modulated by top‐down factors such as pitch predictability, musical expertise, and enjoyment. In addition to the rhythm, musical pitch surprisal is tracked for both low and high predictable music. This supports the view that long‐term musical pitch predictability is processed in the brain and used to facilitate rhythm processing. For tonal, more predictable, music, the ability to make valid pitch predictions seems to facilitate the ability to behaviorally follow the rhythm. For atonal music, the reduced pitch predictability results in stronger acoustic envelope tracking than for tonal music, possibly related to higher prediction errors. These higher prediction errors also seem to come at the cost of finger‐tapping performance, as individuals with stronger pitch surprisal tracking show worse behavioral rhythm tracking. Overall, our findings indicate that rhythm processing interacts with nonrhythmic stimulus properties, in our case pitch surprisal, and listeners’ characteristics such as music expertise and enjoyment.

## AUTHOR CONTRIBUTIONS

A.K.: Conceptualization, methodology, software, validation, formal analysis, investigation, writing—original draft, writing—review and editing, visualization, supervision, funding acquisition. C.P.: Resources, writing—original draft. X.G.: Resources. E.W.: Investigation. L.W.: Conceptualization, investigation. S.A.: Investigation. I.M.: Writing—original draft. C.K.: Methodology, writing—review and editing. J.R.: Investigation, methodology, resources, writing—review and editing.

## COMPETING INTERESTS

The authors have no competing interests.

### PEER REVIEW

The peer review history for this article is available at: https://publons.com/publon/10.1111/nyas.15315

## Supporting information



Supporting Information

## Data Availability

Data and stimuli are publicly available on the OSF (https://osf.io/3gf6k/).
