# Supplementary material for: Cortical and behavioral tracking of rhythm in music: Effects of pitch predictability, enjoyment, and expertise
Source: Ann N Y Acad Sci. 2025 Mar 18;1546(1):120–35. doi: 10.1111/nyas.15315 (PMC11998481; doi:10.1111/nyas.15315)
Supplement: Supplementary file 1 — Supporting Information [file NYAS-1546-120-s001.docx]

# Supplemental analyses

1. **Conditional MI analysis**

To explore whether pitch intervals influence acoustic envelope tracking (and our result that envelope tracking is stronger in the atonal than the tonal condition), we performed a conditional MI analysis (Brohl *et al.*, 2022; Ince *et al.*, 2017). Here, all parameters were identical to the main analysis of acoustic envelope tracking (**Figure 5A**), but we included pitch intervals as the to-be-partialled-out signal in both tonal and atonal conditions.

To extract pitch values, we first used the MIDI toolbox (Eerola & Toiviainen, 2004) for melody and bass lines of the tonal and atonal conditions. The difference between subsequent MIDI pitch values was then used to obtain a measure of pitch intervals, and to create a continuous signal where each pitch interval was as long as the corresponding note (equivalent to how the surprisal signal was created). These pitch interval signals were then normalised using Gaussian Copulas, before including them in the conditional MI analysis. This means each MI computation (for tonal and atonal conditions) included the EEG signal, the envelope signal, and the to-be-partialled out pitch interval signals for both the melody and bass lines.

With pitch intervals in melody and bass lines partialled out, the tracking of the acoustic envelope yielded similar results as the initial tracking analysis (**Figure S1A**). When compared against chance, the envelope was tracked significantly in both tonal and atonal conditions. In the tonal condition, we found a large positive cluster of 31 electrodes that tracked amplitude fluctuations significantly (*p* = .002, *MI*_sum_ = 0.325). Equivalently, in the atonal condition, there was a positive cluster of 32 electrodes that showed significant envelope tracking (*p* < .002, *MI*_sum_ = 0.430). A direct comparison showed that envelope in the atonal condition was tracked stronger than in the tonal condition in two clusters, one over frontal electrodes (*p* = .002, Cohen’s *d*_peak_ = -1.21, 6 electrodes) and one over left occipital electrodes (*p* = .002, Cohen’s *d*_peak_ = -2.17, 3 electrodes). This suggests that, with pitch intervals partialled out, the acoustic envelope is still tracked above chance level, and this tracking is still stronger in the atonal condition.


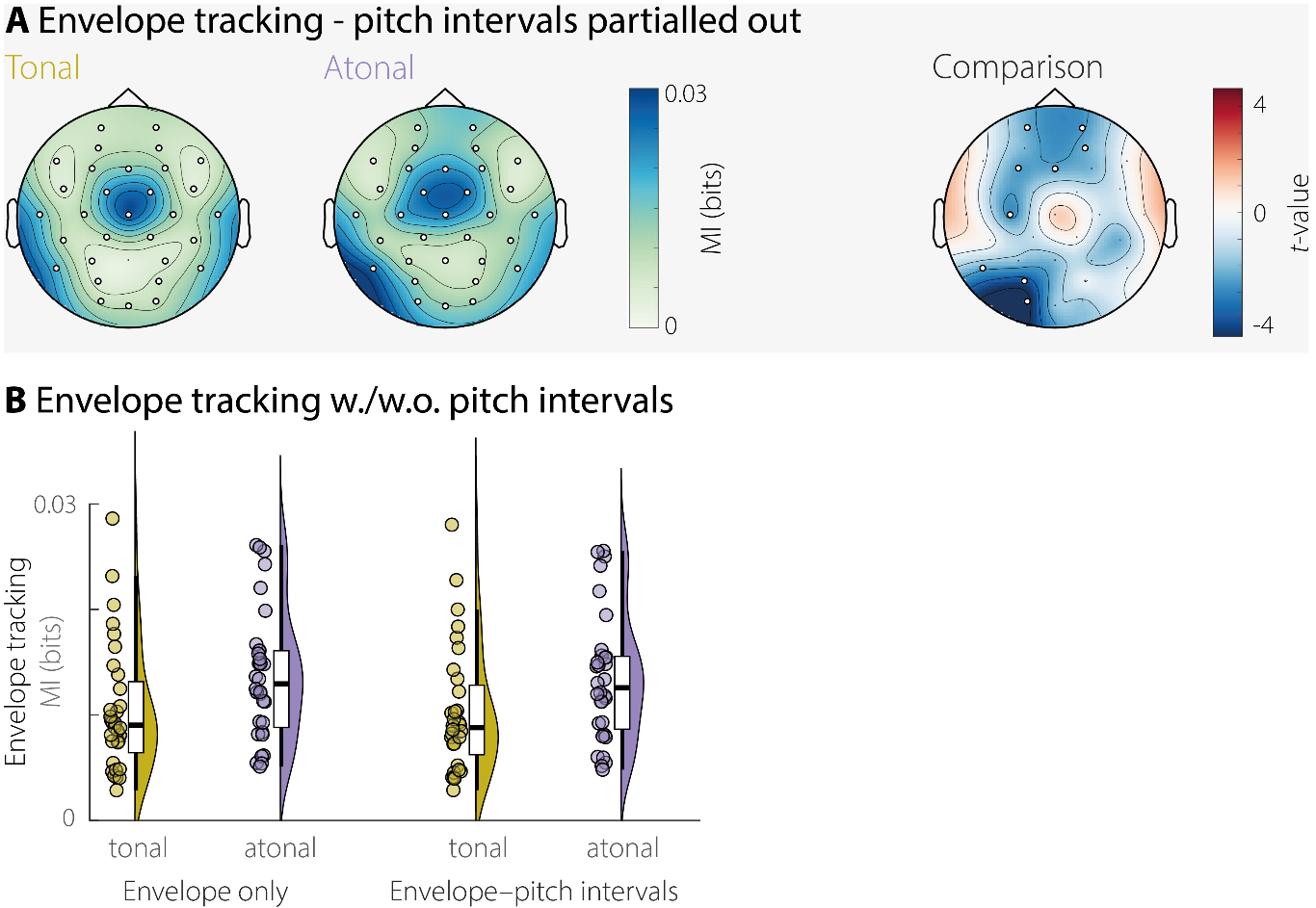


**Figure S1. Conditional MI analysis of envelope tracking with pitch intervals partialled out. A)** Topography of cortical envelope tracking assessed through mutual information (in bits) for both conditions, with partialled out pitch intervals in the melody and bass lines. The right topography shows *t*-values from a direct comparison between tonal and atonal music. Significant electrodes are highlighted with white circles. **B)** Envelope tracking for all electrodes, averaged across participants, for the tonal and atonal conditions, and for the original MI analysis (tracking of envelope) and the conditional MI analysis (tracking of envelope, pitch intervals partialled out). MI values are systematically lower when pitch intervals are partialled out, but this effect is not different for the tonal or atonal condition.

To directly compare MI values across tracking analyses (with and without pitch intervals partialled out), we averaged MI values for each electrode across participants (**Figure S1B**) and performed a repeated measures 2x2 ANOVA with factors *tracking measure* (envelope tracking vs conditional envelope tracking) and *tonality* (tonal vs atonal). Both main effects were significant (*tracking* *measure*: *F*(1,31) = 129.93, *p* < .001, η_p_^2^ = .807; *tonality*: *F*(1,31) = 31.39, *p* < .001, η_p_^2^ = .503). The MI values were lower when the tracking of pitch intervals was partialled out (*tonal*: *M*_Env_ = 0.011, SD = 0.006, *M*_Env-Pitch_ = 0.010, SD = 0.006; *atonal*: *M*_Env_ = 0.014, SD = 0.006, *M*_Env-Pitch_ = 0.013, SD = 0.006). MI values were higher in the atonal than the tonal condition, reflecting the difference shown in **Figure S1A**. Crucially, the interaction *tracking measure × tonality* was not significant (*F*(1,31) = 1.51, *p* > .227, η_p_^2^ = .047), which implies that pitch-interval tracking did not affect the conditions differently.

In sum, while there is a small but highly consistent decrease in MI when the tracking of pitch intervals is partialled out, this effect is not different in tonal and atonal conditions. Our previous finding that the acoustic envelope in the atonal condition is tracked stronger than in the tonal condition, therefore also holds true in the conditional MI analysis. This means that the found differences in envelope tracking are unlikely to be due to low-level differences in the tracking of pitch intervals.

1. **Note onset surprisal**

To confirm that the timing of notes was identical across tonal and atonal music excerpts, we analysed onset surprisal of both conditions using IDyOM, analogous to the analysis of pitch surprisal (see “Pitch surprisal modelling” in manuscript). Results show that onset surprisal was indeed identical in both conditions (**Figure S2**; melody line – tonal: *M*= 4.57 bits, *SD*= 3.14 bits vs. atonal: *M*= 4.57 bits, *SD*= 3.14 bits; bass line – tonal: *M*=4.29 bits, *SD*=2.72 bits vs. atonal: *M*=4.29 bits, *SD*=2.29 bits).


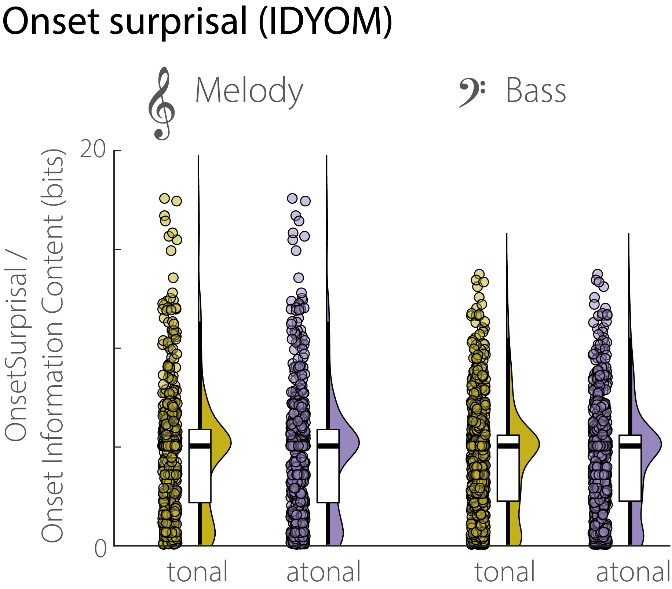


**Figure S2. Onset surprisal in the tonal and atonal conditions.** Onset surprisal (quantified through onset information content) values for each note in the melody and bass lines of both tonal and atonal 5-min excerpts. Points indicate data for all notes, violin plots show kernel density estimates, and boxplots show median interquartile ranges and minimum/maximum. Onset surprisal was identical in both conditions.

**References**

Brohl, F., Keitel, A., & Kayser, C. (2022). MEG Activity in Visual and Auditory Cortices Represents Acoustic Speech-Related Information during Silent Lip Reading. *eNeuro*, *9*(3). <https://doi.org/10.1523/ENEURO.0209-22.2022>

Eerola, T., & Toiviainen, P. (2004). *MIDI Toolbox: MATLAB Tools for Music Research*. University of Jyväskylä.

Ince, R. A., Giordano, B. L., Kayser, C., Rousselet, G. A., Gross, J., & Schyns, P. G. (2017). A statistical framework for neuroimaging data analysis based on mutual information estimated via a gaussian copula. *Hum Brain Mapp*, *38*(3), 1541-1573. <https://doi.org/10.1002/hbm.23471>
